# Moths versus Bees: Contrasts in Habitat Preferences Across Barrens of the Northeastern USA

**DOI:** 10.1002/ece3.70533

**Published:** 2024-11-13

**Authors:** Andrew M. Barton, Helen M. Poulos, Elizabeth Crisfield, Amanda Dillon, Mark Mello, Jennifer Selfridge, Rick Van de Poll, Sarah Hardy

**Affiliations:** ^1^ Department of Biology University of Maine at Farmington Farmington Maine USA; ^2^ Department of Earth and Environmental Sciences and the Bailey College of the Environment Wesleyan University Middletown Connecticut USA; ^3^ Strategic Stewardship Initiative State College Pennsylvania USA; ^4^ New York State Department of Environmental Conservation Albany New York USA; ^5^ Lloyd Center for the Environment Dartmouth Massachusetts USA; ^6^ Maryland Department of Natural Resources United States, Wye Mills Wildlife and Heritage Service Wye Mills Maryland USA; ^7^ Ecosystem Management Consultants of New England Sandwich New Hampshire USA; ^8^ Division of Mathematics University of Maine at Farmington Farmington Maine USA

**Keywords:** barrens, bees, climate, diversity, flowers, moths, Northeast USA, pollinators, sand, species composition

## Abstract

Bees and moths are globally important pollinators. Xeric barrens in the largely mesic northeastern USA support high levels of pollinator diversity, including rare bees and moths. We investigated the response of bee vs. moth communities to abiotic and vegetation drivers in barrens across the region. We sampled local environmental conditions, vegetation, bees, and moths for 2–4 years in 19 preserves. Employing random forest analysis, we tested the role of 29 abiotic and vegetation predictors of bee vs. moth abundance, species richness, Shannon‐Wiener Index, evenness, and species composition. Variables related to climate, canopy cover, and soils were the most important predictors of abundance, diversity, and species composition for both bees and moths. Vegetation variables, such as species richness of shrubs and hostplants, were also important for bees. The direction of these relationships contrasted sharply between bees and moths: bees were more abundant and species rich in more open, sandy sites and moths the opposite. Habitat preferences for a subset of moth xeric specialists were much more similar to bees than to other moths, with a preference for open, sandy conditions. Contrasts between bees and moths in habitat preferences likely stemmed from differences in their life histories: bees rely on flowers for feeding and porous substrates for nesting, whereas most moth adults feed on flowers, but many moth caterpillars use woody plants as hosts. In sharp contrast to the results for abundance and richness, bees and moths responded similarly for the Shannon‐Wiener Index, which raises important general questions about the conservation value of these two metrics. Our results suggest that, because of differences in habitat preferences among pollinators, barrens management for both open and more closed habitats is likely to promote the highest abundance and diversity of local bee and moth pollinator communities jointly.

## Graphical abstract

We investigated the response of bee vs. moth communities to abiotic and vegetation drivers in barrens across the Northeast USA. Bee and moth communities contrasted in their habitat preferences for abundance and species richness, but not for the Shannon‐Wiener Diversity Index. Our results suggest that barrens management for both closed and open canopies is likely to promote the highest abundance and diversity of bee and moth communities jointly.

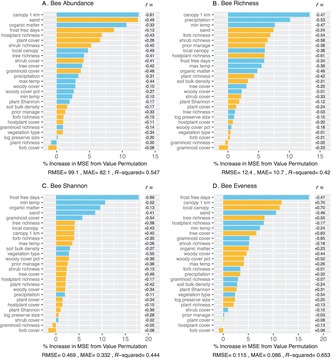

## 1 Introduction

Xeric habitats in largely mesic landscapes are critical reservoirs of biodiversity and rare species (Anderson, Fralish, and Baskin 1999; Andrés and Ojeda 2002; Feeser and O'Connell 2009; Leal, Tabarelli, and da Silva 2003; Pärtel et al. 1999; Proctor and Woodell 1971; Rundel and Palma 2000). In the northeastern United States, for example, barren ecosystems support diverse pollinator communities, including rare species (Bois et al. 2023; Bried, Patterson III, and Gifford 2014; Bried and Dillon 2012; Dunwiddie 1991; Grand and Mello 2004; Jamison, D'Amato, and Dodds 2023; Motzkin and Foster 2002; Thompson et al. 2022; Wagner, Nelson, and Schweitzer 2003). These barrens occur only in highly restricted, dryland sites in an otherwise mesic landscape, on rocky and sandy substrates with excessively well‐drained soils that support sandplain grasslands, heathlands, pine‐oak scrub, and pitch‐pine woodlands. Most are located where glacial processes at the end of the Pleistocene deposited outwash sand or scraped the land to bedrock (Motzkin and Foster 2002). These soils offer limited moisture and nutrients, constraining plant growth and cover and promoting vegetation that readily burns. Before Euro‐American settlement, many of these sites were maintained as barrens rather than developing into denser tree‐dominated vegetation by both lightning‐caused wildfires and intentional Indigenous burning (Abrams and Nowacki 2021; Abrams and Orwig 1995; Anderson, Fralish, and Baskin 1999; Forman 2012; Nowacki and Abrams 2008; Parshall et al. 2003; Poulos 2015; Pyne 2019). Intensive land use by Euro‐American settlers in the 19th and 20th centuries maintained, expanded, and created new openland barren habitats in the Northeast on substrates that were already excessively well‐drained (Copenheaver, White, and Patterson III 2000; Fuller et al. 1998; Jordan, Patterson III, and Windisch 2003; Motzkin, Patterson III, and Foster 1999; Motzkin and Foster 2002).

Regardless of their origin, many previously open barrens have been transitioning to denser, mesophytic vegetation as a result of fire suppression and the decline of intensive land use (Bried, Patterson III, and Gifford 2014; Foster and Motzkin 2003; Jordan, Patterson III, and Windisch 2003; Nowacki and Abrams 2008). Without active reduction in vegetative cover, most contemporary barrens will likely succeed to closed forests, with attendant loss of rare species, including pollinators, that depend on these habitats (Bried, Patterson III, and Gifford 2014; Foster and Motzkin 2003; Jordan, Patterson III, and Windisch 2003; Wagner 2012). The loss of these rare ecosystems has stimulated a concerted effort to better understand and restore extant and former barrens through a mix of treatments, including prescribed fire, thinning, mowing, herbicide application, and planting, aimed at reducing vegetation cover and promoting barren plant species (Bried and Dillon 2012; Jamison, D'Amato, and Dodds 2023; Tucker and Rehan 2019).

Conservation of bee communities is a key rationale for restoring barrens in the Northeast and elsewhere. Most, but not all, bee species in temperate regions have an affinity for open conditions, at least in part because of the higher density of flowers produced by herbaceous and shrub species supported in these areas (Antoine and Forrest 2021; Danforth et al. 2019; Galbraith et al. 2019; Michener 2000; Murray, Kuhlmann, and Potts 2009; Winfree 2010; Winfree, Griswold, and Kremen 2007). Many of these plant species are important host plants for bees, providing abundant nectar and pollen, the primary foods for these taxa (Fowler 2016). Additionally, the majority of bee species are ground nesters, preferentially nesting in more porous, less‐compacted soil with a high percentage of sand, although substantial variation exists among species (Antoine and Forrest 2021; Harmon‐Threatt 2020). These substrate conditions tend to support more open conditions due to their unproductive soils. As a result of the correspondence between bee requirements and barren habitat conditions, bees are generally abundant and diverse in these sites, which are enhanced by active management to decrease woody plant cover (Campbell, Hanula, and Waldrop 2007; Campbell et al. 2018; Lettow et al. 2018; Leuenberger et al. 2016; Ulyshen et al. 2022; Wagner, Nelson, and Schweitzer 2003).

Less appreciated, moths are also diverse and threatened in the Northeast USA (Bartomeus et al. 2013; Schweitzer, Minno, and Wagner 2011; Wagner 2012; Wagner, Nelson, and Schweitzer 2003). Unlike bees, most moth species do not exhibit a clear preference for open conditions, with many preferring forests (Rákosy and Schmitt 2011; Schweitzer, Minno, and Wagner 2011). The role of plant species in moth life history differs from that in bees. The adults of both feed on flowers (bees during the day, moths mainly at night), but whereas bee juveniles depend on adult foraging at flowers as well as appropriate nesting sites, juveniles of most moths feed on foliage or other plant parts. Like the strategies of many other insect herbivores, moth caterpillars often feed on just a single plant species or genus, many of which are shrubs and trees (Bried and Dillon 2012; Maier 2004; Robinson et al. 2024). Thus, the impact of plant species and vegetation structure on moth communities is in part determined by the host‐plant preferences of juveniles in ways that do not occur with bees. Accordingly, moth communities are likely to be less tied to open conditions, sandy soils, and herbaceous and shrub species that provide floral resources than are bees. On the other hand, a subset of moth species in the Northeast have an affinity for open, xeric sites (Schweitzer, Minno, and Wagner 2011; Wagner, Nelson, and Schweitzer 2003). Accordingly, we expect these species to respond in a contrasting manner to the bulk of moth species and behave more like bees in their habitat specificity.

In this paper, we compare the abiotic and vegetation drivers of bee versus moth abundance, diversity, and species composition across 19 barrens in the Northeast USA. These barrens vary considerably in geographic location, climate, soils, and vegetation, despite sharing more xeric conditions than most of the northeastern landscape. We focus on bees and moths because their differences in life histories raise the possibility that management to reduce vegetation cover while promoting bee communities might negatively affect moths. These opposing impacts are not certain, however, both because many barrens support woody plants that serve as moth host plants and some moth species specialize on more open, xeric conditions.

We address three key questions in this paper focused on examining the fundamental responses of bees and moths to abiotic and vegetation variation across northeastern barrens. First, what are the relative roles of climate, soil properties, canopy cover, and vegetation traits in controlling bee and moth abundance, diversity, and species composition? Second, to what extent do bee and moth communities differ in their responses to these abiotic and biotic factors? Third, how are abundance, diversity, and species composition correlated between bees and moths across the landscape? A chief aim of addressing these questions is to illuminate the contrasts between bees and moths in their responses to xeric habitats and the implications for management to conserve both sets of pollinators (deMaynadier et al. 2023; Nath, Singh, and Mukherjee 2023).

## 2 Methods

### 2.1 Study Areas and Design

**FIGURE 1:**
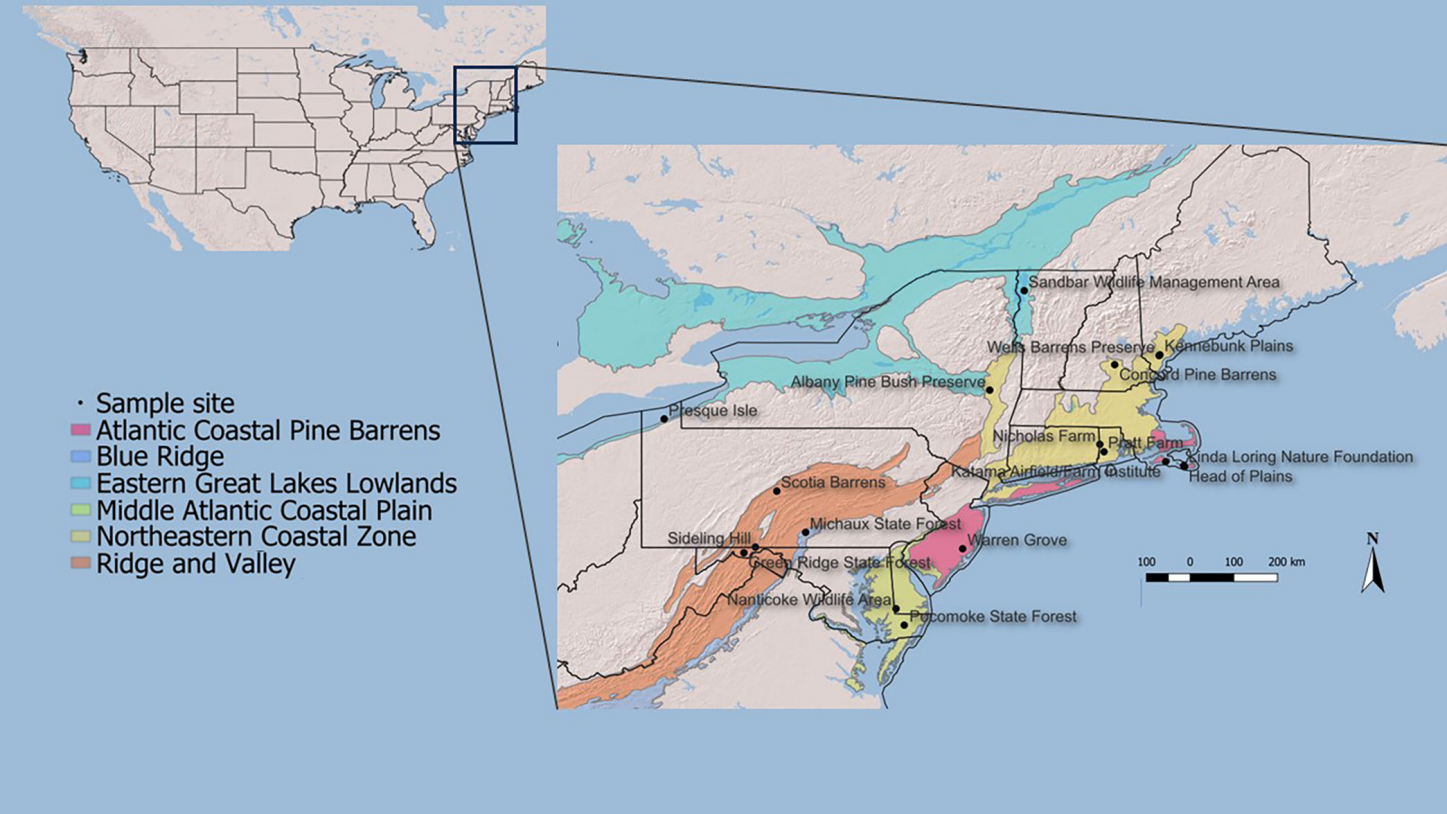
Map showing locations of 18 preserves sampled for some combination of bees, moths, vegetation, and abiotic variables. USA State lines and Environmental Protection Agency Level III ecoregions (Omernik 1987) are provided for geographic and ecological context.

We carried out research in 19 barrens preserves in the Northeast USA (Figure 1, Table 1), which span seven ecoregions across 800 km south to north and 900 km east to west, from Delaware to northern Vermont and from southwestern Maine to western Pennsylvania. These rare sites typically support some combination of tree‐sized oaks (*Quercus* spp.), scrub oak (*Quercus ilicifolia* Wangenh.), pitch pine (*Pinus rigida* Mill.), Ericaceous shrubs (e.g., *Vaccinium* species), other shrubs, and a variety of forbs and graminoids—the local vegetative mix depending on climate, soil conditions, and past land use (Anderson, Fralish, and Baskin 1999; Motzkin and Foster 2002; Raleigh, Capece, and Berry 2003). Collectively, these xeric sites exhibit substantial variation in climate, topography, soils, and vegetation, occurring in ecoregions as different as the Blue Ridge and the Northeastern Coastal Zone. The 19 preserves also vary considerably in ownership, size, mission, management history, and adjacent land use. Similar rare barren sites exist in other parts of North America (Anderson, Fralish, and Baskin 1999), some of which have been studied for bees (e.g., Wolf et al. 2022) and moths (e.g., Shuey, Metzler, and Tungesvick 2012).

**TABLE 1:** Preserves used in the analyses of bee versus moth communities, including preserve name and state, ownership, latitude and longitude, preserve size, amount of barrens area, number of preserve units sampled, mean annual daily temperature, and mean annual precipitation.

Preserve	Ownership	Latitude	Longitude	Preserve size (ha)	Preserve barrens area (ha)	Number of preserve units sampled	Ecoregion^b^	Mean annual daily temp (°C)^c^	Mean annual precip (mm)^c^
Albany Pine Bush Preserve Commission, NY	NYS Benefit Corporation	42.71639	−73.87574	1356	1356	5	Northeastern Coastal Zone	9.2	1039.2
Concord Pine Barrens, NH	State Land	43.19558	−71.49600	121	121	2	Northeastern Coastal Zone	8.6	1109.9
Green Ridge State Forest, MD	State Land	39.61965	−78.56826	19,838	500	1	Ridge and Valley	12.0	996.8
Head of Plains, MA	State Land Bank	41.26449	−70.17403	405	405	3	Atlantic Coastal Pine Barrens	10.7	1185.5
Katama Airfield + Farm Institute, MA	Private	41.35638	−70.52503	197	197	2	Atlantic Coastal Pine Barrens	10.8	1203.4
Kennebunk Plains Preserve, ME	Nature Conservancy	43.40537	−70.62858	55	1296^a^	1	Northeastern Coastal Zone	8.2	1261.2
Linda Loring Nature Foundation, MA	Non‐profit Foundation	41.28957	−70.17791	109	42	2	Atlantic Coastal Pine Barrens	10.6	1188.3
Michaux State Forest, PA	State Land	40.02668	−77.38011	34,615	599	3	Blue Ridge	10.1	1287.5
Nanticoke Wildlife Area, DE	State Land	38.56128	−75.66835	1825	202	2	Middle Atlantic Coastal Plain	14.0	1191.8
Nicholas Farm Management Area, RI	State Land	41.68521	−71.77440	579	149	1	Northeastern Coastal Zone	10.0	1276.6
Pocomoke State Forest, MD	State Land	38.21690	−58.71056	7287	7287	2	Middle Atlantic Coastal Zone	14.2	1193.3
Pratt Farm Conservation and Recreation Area, MA	Town Land	41.55470	−71.70657	65	4	1	Northeastern Coastal Zone	10.5	1272.1
Presque Isle State Park, PA	State Land	42.16606	−80.08609	1259	1259	4	Eastern Great Lakes Lowland	9.7	1006.0
Sandbar Wildlife Management Area, VT	State Land	44.62259	−73.22782	632	632	1	Eastern Great Lakes Lowland	8.0	919.4
Scotia Barrens, PA	State Land	40.80003	−77.94603	2632	2632	3	Ridge and Valley	9.5	1078.7
Sideling Hill Creek Conservation Area, PA	Western Pennsylvania Conservancy	39.75324	−78.36574	104	104	3	Ridge and Valley	11.5	987.1
Warren Grove Recreation Area, NJ	State Land	39.70137	−74.38835	250	250	3	Atlantic Coastal Pine Barrens	12.4	1219.6
Wells Barrens Preserve, ME	Nature Conservancy	43.38022	−70.64234	220	1296^a^	1	Northeastern Coastal Zone	8.2	1259.9

^a^ Including adjacent conservation easement.

^b^ Omernik (1987).

^c^ Climate data are 30‐year normals (1991–2020; PRISM, Northeast Alliance for Computational Science and Engineering, Oregon State University). Accessed [12/10/22]; for preserve units with multiple sample sites, mean climate values are provided.

Table 2 lays out our unbalanced sampling scheme. Two of the 19 preserves, Farm Institute and Katama Airfield, were treated as a single preserve for analysis because they were contiguous and supported similar habitat, reducing the number of preserve locations to 18. We sampled bees in all 18 and moths in 16 of the preserves. In 12 preserves, we sampled in multiple ‘preserve units’, which corresponded with already‐existing management compartments, most of which captured ecological variation across the property. In six preserves, we sampled in only one location. For bees, sampling was carried out in one sample site per preserve unit; for moths, sampling was carried out in 1–3 sample sites per preserve unit. In total, we quantified bees in 39 sample sites and moths in 45. Abiotic data were recorded or acquired for all preserve units; where multiple sample sites occurred for a preserve unit (moths only), abiotic data for the entire preserve unit were used for each sample site. We surveyed vegetation in a total of 147 transects in 31 of the 39 bee sample sites (in 14 preserves) and 25 of the 45 moth sample sites (in 13 preserves) in close proximity to bee and moth sample sites. The number of vegetation transects per sample site varied from 1 to 20. Sampling was repeated for 2–4 years (2018–2021) for bees, 2 years (2021–2022) for moths, and 2–5 years (2018–2022) for plants. Only native bees and moths, which could be identified to species, were included in the data sets analyzed herein.

**TABLE 2:** Number of sample sites and years of sampling for bees, moths, and plants.

Preserve	Preserve unit	Bee sample sites	Bee sample years	Moth sample sites	Moth sample years	Plant transects	Plant sample years
Albany Pine Bush	Alley Cat	1	2019–2021	1	2021–2022	13	2019–2021
Albany Pine Bush	Bivy	1	2019–2021	1	2021–2022	20	2019–2021
Albany Pine Bush	Diversity	1	2019–2021	ns	2021–2022	20	2019–2021
Albany Pine Bush	Draperies	1	2019–2021	ns	2021–2022	20	2019–2021
Albany Pine Bush	Greentaxis	1	2019–2021	1	2021–2022	10	2019–2021
Concord Pine Barrens	East	1	2019, 2021	3	2021–2022	8–11	2019–2022
Concord Pine Barrens	North	1	2019–2021	ns	ns	ns	ns
Green Ridge State Forest	Town Creek	1	2018–2021	3	2021–2022	4	2019–2021
Head of Plains	Barrett Farm Road	1	2018–2021	1	2021–2022	1	2018–2021
Head of Plains	Red Barn Road	1	2018–2021	1	2021–2022	1	2018–2021
Head of Plains	Sheep Pond Road	1	2018–2021	1	2021–2022	1	2018–2021
Katama Airfield	Katama Airfield	1	2018–2021	3	2021–2022	3	2018, 2020–2021
Katama Airfield	Farm Institute	1	2018–2020	ns	ns	ns	ns
Kennebunk Plains	Kennebunk Plains	1	2018, 2020, 2021	3	2021–2022	ns	ns
Linda Loring	Beach Plum	1	2018–2021	1	2021–2022	1	2018–2021
Linda Loring	Osprey Pole	1	2018–2021	2	2021–2022	1	2018–2021
Michaux State Forest	Dead Woman Hollow	1	2020–2021	1	2021–2022	4	2020–2022
Michaux State Forest	Hogshead Road	1	2020–2021	1	2021–2022	4	2020–2022
Michaux State Forest	Woodrow Road	1	2020–2021	1	2021–2022	4	2020–2022
Nanticoke Wildlife Area	Phillips Landing Lupine	1	2020–2021	3	2021–2022	1	2020–2021
Nanticoke Wildlife Area	Phillips Landing Ridge	1	2020–2021	ns	2021–2022	1	2020–2021
Nicholas Farm	Nicholas Farm	1	2020–2021	2	2021–2022	4	2020–2022
Pocomoke State Forest	Foster Tract	1	2018–2021	1	2021–2022	2	2019–2021
Pocomoke State Forest	Furnace Tract	1	2018–2021	2	2021–2022	7	2019–2021
Pratt Farm	Pratt Farm	1	2020–2021	1	2021–2022	3–4	2020–2022
Presque Isle	Beach 10	1	2020–2021	ns	ns	2	2020–2022
Presque Isle	Beach 11	1	2020–2021	ns	ns	3	2020–2022
Presque Isle	Dead Pond Trail E	1	2020–2021	ns	ns	3	2020–2022
Presque Isle	Dead Pond Trail W	1	2020–2021	ns	ns	ns	ns
Sandbar Wildlife Area	Sandbar Wildlife Area	1	2018–2021	ns	ns	ns	ns
Scotia Barrens	Ore Pit Pond	1	2020–2021	1	2021–2022	5	2020–2022
Scotia Barrens	Ore Wash Barrens	1	2019–2021	1	2021–2022	3	2020–2022
Scotia Barrens	Ten Acre Pond	1	2019–2021	1	2021–2022	4	2020–2022
Sideling Hill	Nose	1	2020–2021	1	2021–2022	1–2	2020–2022
Sideling Hill	Pine	1	2020–2021	1	2021–2022	2	2020–2022
Sideling Hill	Rock	1	2020–2021	1	2021–2022	2	2020–2022
Warren Grove	NJ02	1	2020–2021	1	2021–2022	ns	ns
Warren Grove	NJ03	1	2020–2021	1	2021–2022	ns	ns
Warren Grove	Range	Ns	ns	1	2021–2022	ns	ns
Wells Barrens Preserve	Wells Barrens Preserve	1	2018, 2020–2021	3	2021–2022	ns	ns
Total		39		45		147	

*Note:* Each bee site was a transect with data summed across 24 bee bowls; each moth site was a single bucket; each plant site was a transect.

Abbreviation: ns, not sampled.

### 2.2 Bee Surveys

Bees were destructively sampled via bee bowls (pan‐trapping), protocols loosely based on LeBuhn et al. (2003) and adapted from the Very Handy Bee Manual (Droege 2015; Droege et al. 2010). This sampling approach had no negative impacts on bee populations in the one study where it was examined previously (Gezon et al. 2015), but tradeoffs exist between this and alternative bee monitoring approaches (Portman, Bruninga‐Socolar, and Cariveau 2020; Prendergast et al. 2020). There are also potential biases in pan trapping depending on floral resources (Baum and Wallen 2011). Bee bowls consisted of standard plastic 3.25 oz. souffle cups painted with blue, yellow, and white ultraviolet paints and filled halfway with water and non‐citrus detergent. The color of the bowls attracts bees, while the soap breaks the surface tension of the water, allowing for the lethal capture of bee specimens.

At each of the 39 bee sample sites, for each sample period, we employed 24 bee bowls placed on the ground 5–10 m apart, aligned along a transect. Transect start and end points were permanently marked in the field or with a GPS unit so that the same transect could be resampled over the growing season and from year to year. Transects were situated so that they were at least 30 m from habitat edges. Bee bowls were installed before 9 a.m. and removed by 5 p.m. the same day, or alternatively put in place after 5 p.m. the day before and left out for no more than 24 h. For each sample site, all captured specimens were pooled across the 24 bee bowls for each sampling period, strained, and placed in labeled whirl‐pak bags with 70% ethanol.

Sampling was conducted during five 2‐week windows between May and September (May 1–14, June 3–17, July 1–23, August 5–19, and September 10–24) from 2018 to 2021. In order to standardize for bee activity, sites were sampled only on calm, sunny days (> 90% sun) with air temperatures above 20°C. These conditions maximize the activity of bees, as they are generally not active under cool or rainy conditions and only forage once they have sufficiently warmed (Roberts and Harrison 1998). We only included site‐years in our analysis that were sampled at least during four of the sample windows within a given year. Samples for a given year were summed across sample periods for each site.

Bee bowls are not specific to bees and catch a wide variety of insects, including flies, butterflies, moths, wasps, beetles, etc. Some sample sites in the project had rare or protected insects (e.g., Frosted elfin, *Callophyrus irus* (Godart, [1824]), Lycaenidae; Karner blue, *Lycaeides melissa samuelis* (W.H. Edwards), Lycaenidae); accordingly, we did not sample during their flight periods to minimize impacts on these taxa unless we first procured permission from the appropriate management agencies.

In 2018, all bees were mailed to Joan Milam for identification at the University of Massachusetts. In other years, Sam Droege, Clare Maffei, and Sydney Shumar sorted, pinned, and identified the specimens to the species level (when possible) at the USGS Native Bee Inventory and Monitoring Lab at the Patuxent Wildlife Research Center in Laurel, Maryland. Specimens were identified to the species level and registered by Hodges number using the bee species identification section of Discover Life (Ascher and Pickering 2024). Identification was carried out independently by two taxonomists, and bees unidentified by one person were examined by the other. Voucher specimens were not collected for bees, but bees were made available to preserves and other insect research teams.

### 2.3 Moth Surveys

We sampled nocturnal moth diversity and abundance at each of the 45 moth sample sites from April to September in 2021 and 2022, with specific dates determined by weather, moon phase, schedule constraints, and other factors. Most preserves were sampled five times during each year; northernmost sites were sampled three times due to the shorter season. We included in our analyses only site‐years that were sampled at least during three of the sample windows within a given year. Samples for a given year were summed across sample periods for each site.

We destructively sampled moths using one 15‐W UV light bucket trap per sample site, set at 1 m height, armed with an appropriate killing agent (e.g., acetone or ethyl acetate). Bucket traps were placed before sunset and the contents collected after sunrise the following day. Lamps were powered by car batteries, and light sensors were used to turn on lamps as needed given site logistics. Collection buckets were co‐located with bee bowls. In preserve units where moths were sampled at more than one sample site, collection buckets were placed at least 100 m apart. To maximize collection, traps were set on nights with no or little predicted rainfall, low wind speed, high temperature, high humidity, and near a new moon or with heavy cloud cover to limit moonlight. Conditions were recorded for each collection event.

We identified Macrolepidoptera (SuperFamilies Drepanoidea, Geometroidea, Mimallonoidea, Bombycoidea, Sphingoidea, and Noctuoidea) and three Microlepidoptera families (Limacodidae, Crambidae, and Tortricidae) to species level (when possible), with the aid of Beadle and Leckie (2012), Covell Jr. (2005), and Nanz (2024), and referenced specimens to Hodge's checklist (Hodges 1983) and Pohl's phylogenetic sequence numbers (Pohl, Patterson, and Pelham 2016). Voucher specimens for sites in Maine and New Hampshire were deposited at the University of New Hampshire Zoological Museum; all others were sent to the Biodiversity Research Collection at the University of Connecticut. Only newly‐documented species for this project were vouchered in 2022.

### 2.4 Vegetation Surveys

We sampled vegetation during the peak of the growing season, between mid‐July and mid‐August to ensure that plants were leafed out for identification. To ensure that repeated sampling occurred in the same place, GPS locations were taken with high‐quality units or plots were permanently monumented. Beginning at a previously selected random point, a 50‐m transect was run to the north for each vegetation sample location. In steep locations, transect lines were laid across the slope contour and the line azimuth for each was recorded.

We sampled plant cover via line‐point intercept sampling at 50 points along the transect at 1‐m horizontal intervals by dropping a pin to the ground from a standard height and recording the species identity of every plant species intersected by the pin or projected visually for strata > 2 m height. Plant encounters were divided into five strata: ground, 0–1, 1–2, 2–5, and > 5 m, which we then summed over all strata to determine total cover by species at each line‐point intercept point (0%–500% range). A range pole or laser rangefinder was used to identify strata. Each species was recorded only once within each stratum but could be recorded in multiple strata. For each species, we recorded the plant lifeform as forb, graminoid, woody vine, woody shrub, and woody tree. These data were used to calculate total plant cover across all strata for all plants, tree life‐form, shrub life‐form, forb life‐form, graminoid life‐form, woody (trees + shrubs), and woody percentage (woody cover/total cover). These cover estimates are distinct from the remotely‐sensed canopy cover data described below and in Table 3.

**TABLE 3:** Predictor variables are used in random forest (RF) and non‐metric multidimensional scaling (nMDS) analyses.

Predictor	RF models/nMDS	Source	Description	Resolution
Precipitation	Both RF and nMDS	PRISM Climate Group^a^	Mean annual precipitation for 1991–2020	4 km
*T*_min_	Both RF and nMDS	PRISM Climate Group	Mean low daily temperature for 1991–2020	4 km
*T*_max_	Both RF and nMDS	PRISM Climate Group	Mean high daily temperature for 1991–2020	4 km
Frost‐free days	Both RF and nMDS	Daymet^b^	Mean annual number of frost‐free days for 2018–2022	1 km
Sand	Both RF and nMDS	SSURGO Web Soil Survey^c^	Percent sand in soil on a dry mass basis	6–32 m
Organic matter	Both RF and nMDS	SSURGO Web Soil Survey	Percent organic matter in soil on a dry mass basis	6–32 m
Soil bulk density	Both RF and nMDS	SSURGO Web Soil Survey	Dry kgs of soil per m^3^ volume	6–32 m
Local canopy cover	Both RF and nMDS	Multi‐Resolution Land Characteristics (MRLC) Consortium^d^	Remotely‐sensed percent canopy cover at sample site	30 m
1 km canopy cover	Both RF and nMDS	Multi‐Resolution Land Characteristics (MRLC) Consortium	Zonal mean remotely‐sensed percent canopy cover within 1 km of sample site	30 m
Preserve size	Both RF and nMDS	Preserve records, GIS	Preserve area in ha	NA
Prior management	Both RF and nMDS	Preserve staff and records	Active management reducing vegetation cover prior to 2018	Preserve sample site
Vegetation type	Both RF and nMDS	Vegetation sampling	Cluster analysis of vegetation data	50 m (transect)
Plant cover	Expanded RF and nMDS only	Vegetation sampling	Percent total plant cover from field sampling	50 m (transect)
Plant species richness	Expanded RF and nMDS only	Vegetation sampling	Calculations from vegetation data	50 m (transect)
Plant Shannon‐Wiener Index	Expanded RF and nMDS only	Vegetation sampling	Calculations from vegetation data	50 m (transect)
Total woody plant cover	Expanded RF and nMDS only	Vegetation sampling	Calculations from vegetation data	50 m (transect)
Percentage woody plant cover (of total)	Expanded RF and nMDS only	Vegetation sampling	Calculations from vegetation data	50 m (transect)
Total tree cover	Expanded RF and nMDS only	Vegetation sampling	Calculations from vegetation data	50 m (transect)
Total tree species richness	Expanded RF and nMDS only	Vegetation sampling	Calculations from vegetation data	50 m (transect)
Total shrub cover	Expanded RF and nMDS only	Vegetation sampling	Calculations from vegetation data	50 m (transect)
Total shrub species richness	Expanded RF and nMDS only	Vegetation sampling	Calculations from vegetation data	50 m (transect)
Total forb cover	Expanded RF and nMDS only	Vegetation sampling	Calculations from vegetation data	50 m (transect)
Total forb species richness	Expanded RF and nMDS only	Vegetation sampling	Calculations from vegetation data	50 m (transect)
Total graminoid cover	Expanded RF and nMDS only	Vegetation sampling	Calculations from vegetation data	50 m (transect)
Total graminoid species richness	Expanded RF and nMDS only	Vegetation sampling	Calculations from vegetation data	50 m (transect)
Bee host plant cover	Expanded RF and nMDS only	Vegetation sampling	Calculations from vegetation data	50 m (transect)
Bee host plant species richness	Expanded RF and nMDS only	Vegetation sampling	Calculations from vegetation data	50 m (transect)
Moth host plant cover	Expanded RF and nMDS only	Vegetation sampling	Calculations from vegetation data	50 m (transect)
Moth host plant species richness	Expanded RF and nMDS only	Vegetation sampling	Calculations from vegetation data	50 m (transect)

*Note:* Response variables are described in the text. Table shows predictor name, in which model the predictor is used, data source, description, and spatial resolution of the data source. The models are the base and expanded RF models. Base = predictor variables excluding field‐collected vegetation variables; expanded = all predictor variables.

^a^ PRISM, Northeast Alliance for Computational Science and Engineering, Oregon State University. Accessed [12/10/22]; Daly and Bryant (2013).

^b^ Daymet: Annual Climate Summaries on a 1‐km Grid for North America, Version 4 R1, 10.3334/ORNLDAAC/2130. Accessed [12/10/2022]; Thornton et al. (2020).

^c^ Soil Survey Staff (2022), Natural Resources Conservation Service, United States Department of Agriculture. Web Soil Survey. https://websoilsurvey.nrcs.usda.gov/. Accessed [11/10/2022].

^d^ Multi‐Resolution Land Characteristics (MRLC) Consortium. Soil Survey Staff (2022). Accessed [11/19/2022]; Jin et al. (2023).

We generated a list of bee and moth host plants (at the generic level) found in our plant inventory. Host plants were determined using expert knowledge by the bee and moth sampling teams and through literature investigations. We ignored bee and moth species with fewer than 50 total occurrences across all sample sites and years. We compiled this host plant list in order to examine the importance of host‐plant cover in bee and moth abundance, diversity, and species diversity.

### 2.5 Predictor Variables

Two groups of predictor variables were quantified and used in the analyses described below (Table 3). First, from the vegetation surveys described previously, we extracted 18 plant cover and diversity variables. These data were available for 30 (out of 39) bee sample sites and 25 (out of 45) moth sample sites where vegetation was surveyed (Tables 2 and 5).

Second, for all 39 bee and 45 moth sample sites, we obtained data for 11 variables related to climate, soil, canopy cover, preserve size, and past vegetation management (Table 3). Where appropriate, we extracted and derived these data in a geographic information system using the point sampling tool in QGIS (QGIS.Org 2023). We downloaded 30 year climate normals (1991–2020) for mean annual precipitation, mean annual high daily temperature, and mean annual low daily temperature for each sample site from PRISM (Daly and Bryant 2013) and mean annual growing season length for 2018–2021 from Daymet (Thornton et al. 2020). We characterized sample site soils using the SSURGO Web Soil Survey (Soil Survey Staff 2022) tool‐derived estimates, selecting percent sand, soil bulk density, and organic matter—variables likely to have important effects on bees. These data were extracted for a 100‐ha buffer area with the sample coordinates as a center point. Each of these areas comprised 1–16 soil map units, each with unique characteristics. Summary data for each area is the weighted average across map units on an area basis. We extracted 30‐m resolution, remotely‐sensed canopy cover data for each location from the Multi‐resolution Land Characteristics Consortium (Jin et al. 2023). We used these cover data to also calculate the zonal mean canopy cover for the area within 1 km of each sample site. Preserve size was determined either from information provided by the preserve or from shapefiles of preserve boundaries in QGIS. We coded sample sites as experiencing prior management for open conditions or not based on queries of preserve managers.

### 2.6 Statistical Analyses

Tables 2 and 5 show the number of preserves, preserve units, sample sites, and years for bees, moths, and plants used in the statistical analyses, all of which were implemented in R (R Core Team 2023). As described in detail below, we implemented separate analyses of bees and moths and joint analyses of the two groups. Sample sites were sufficiently separated spatially to ensure independent samples of bee and moth communities. However, the spatial distribution of sample sites was not accounted for in the separate or joint analyses. This might have affected the outcomes in at least two ways. First, preserves with multiple preserve units (12 preserves) likely had a larger influence on the results than those with only a single preserve unit (6 preserves). Second, since the geographic sampling was not random or intentionally stratified, the concentration of reserves in some regions and not others could have influenced the impacts of predictors, such as climate, in the random forest models.

#### 2.6.1 Separate Analyses of Bees And Moths

We carried out three sets of statistical analyses in which bees and moths were analyzed separately. First, we investigated the correlation patterns within bees and moths among four dependent variables: abundance, species richness (*S*), Shannon‐Wiener Index (*H*), and evenness (*J*). Abundance is the total number of bees or moths at a sample site for a given year. Species richness is the number of distinct bee or moth species identified at a sample site. The Shannon‐Wiener Index (*H*) is a commonly used diversity metric that reflects both the number of species and the relative abundance of species. Numerically, the index estimates the degree of uncertainty of predicting the species of any randomly‐selected member of the population and is calculated with the formula *H*′ = − ∑ π (ln π), where π is the proportion of individuals in each species. Evenness is a descriptive measure focused on the relative abundance of bees or moths at a sample site. We used Pielou's Index of Evenness (Pielou, 1966), calculated as *J* = *H*/max(*H*) = *H*/ln(*S*), which ranges from 0 to 1, with low evenness occurring where few species numerically dominate the community. These four dependent variables were computed using the R package vegan (Oksanen et al. 2022). We expected species richness to increase with abundance, a near universal pattern (Gaston, 2000). The typical positive relationships of abundance with richness mean that increasing abundance usually leads to higher *H*; on the other hand, there is no general relationship between abundance and evenness. Thus, we did not have a priori expectations for the relationships among abundance, evenness, and *H*.

Second, we used random forest models (Breiman 2001; randomForest in R, Liaw and Wiener 2002) to identify key predictors of variation in abundance and diversity of bee and moth communities, analyzed separately. The purpose of these analyses was both to improve our understanding of important environmental factors acting on bee and moth abundance and diversity and to compare species‐environment relationships between these two groups of pollinators. We chose random forest models because of the unbalanced nature of our data, the lack of distributional assumptions in these models, and the capacity of this analytical approach to model the complex nature of interactions between variables.

We used abundance, species richness, evenness, and *H* as response variables in the random forest models. Because vegetation was not surveyed for all sample sites, we faced a tradeoff regarding which predictors to include. For all sample sites, we had data for four climate variables, three soil variables, two canopy cover variables, preserve size, and previous management for open conditions. Only 30 of 39 bee and 25 of 45 moth sample sites (Table 2) also had data for 18 vegetation predictors (Table 3). Consequently, we ran two types of random forest models each for bees and moths: “base models” that included all sample sites but excluded vegetation predictors and “expanded models” that included all predictors but for a smaller sample size (Table 5). We present the importance of predictors only for the best of these two models for each set of pollinators—expanded models for bees and base models for moths. In initial random forest model runs, we found that year was not an important predictor, and we eliminated this factor by taking the means for dependent variables across years.

We assessed the performance of random forest models using root mean square error, mean absolute error, and *R* ^2^. The relationships between response variables and the most important predictors were further explored using bivariate plots showing the pattern of these relationships. For each random forest model, we show these plots for the six most important predictors (i.e., those where a permutation of the values resulted in the highest increase in percent mean square error).

Because the random forest models revealed a strong affinity of moths for closed forest conditions, we ran separate random forest models for moth species thought to be tied to xeric conditions (69 of the 721 species), testing the hypothesis that this small subset of species would respond differently than the entire set of moth species (Table S1). We did not run a similar analysis for bees because the random forest models pointed to a strong affinity for open conditions for these species. To assemble the xeric moth list, we started with information from published sources for moths potentially associated with xeric habitats in the eastern U.S. (Leuenberger et al. 2016; Schweitzer, Minno, and Wagner 2011; Wagner, Nelson, and Schweitzer 2003). We added other moth species to this list based on expert knowledge of the moth sampling crews, members of which have sampled moths across the Northeast and have been involved in efforts to identify moths associated with uncommon habitats in the region.

In a third set of analyses, we examined relationships of species composition with environment in bees and moths separately using non‐metric multidimensional scaling (nMDS) of species abundances, implemented in the R package vegan (Oksanen et al. 2022). Species composition data are multivariate (species × sample sites), and nMDS is a standard approach for analyzing such data. These analyses can be viewed as parallel to the previously discussed random forest analyses, which were used for the four univariate response variables. Bray‐Curtis distances were used in nMDS to identify the first two axes of gradients in species composition. We then analyzed the associations of predictor variables with site (i.e., sample site) scores using the envfit function in the vegan package. To match the random forest models, we used the expanded models for bees (all predictor variables) and the base models for moths (without the field‐derived vegetation predictors).

#### 2.6.2 Joint Analyses of Bees and Moths

We examined whether bee and moth communities were correlated with each other in the subset of 30 sample sites where both sets of pollinators were sampled. We further restricted the analysis to 2021, the only year in which both bees and moths were sampled. The patterns revealed herein might well differ in other years with different weather and floral conditions. As described previously, we derived total abundance, species richness, evenness, Shannon‐Wiener Index (*H*), and species composition for each sample site separately for bees and moths. Then, we carried out simple linear correlation analyses between the two pollinator groups for all dependent variables, testing the null hypotheses that bees and moths were not associated with these metrics. In the graphs that accompany the results of these correlations, we indicate the degree of canopy cover and soil sand percentage for each sample site to provide insight into the drivers of the community patterns within and between bees and moths.

## 3 Results

### 3.1 Summary Statistics for Abundance and Species Richness

We included a total of 20,115 bees across 173 species for our analyses. The mean number of bees captured per sample site per year was 178.0 (SE = 16.0), with a range of 1–723. The mean for bee species richness was 13.6 (SE = 0.8), with a range of 1–39. We included a total of 25,700 moths across 921 species for our analyses. The mean number of moths captured per sample site per year was 535.4 (SE = 60.9), with a range of 83–1994. The mean for moth species richness was 98.6 (SE = 6.5), with a range of 33–213.

### 3.2 Separate Analyses of Bees and Moths

#### 3.2.1 Correlations Among Dependent Variables

The relationships among abundance, species richness, evenness, and Shannon‐Wiener Index (*H*) were similar for bees and moths in some cases but notably different in others (Table 4). For both bees and moths, there was a significant positive correlation between abundance and richness, as expected, and between *H* and evenness. For moths there was a significant positive correlation between richness and *H*, while for bees, the correlation, while also positive, was not significant. The correlation between abundance and *H* was significantly positive for moths but, in stark contrast, significantly negative for bees. The correlations between abundance and evenness and between richness and evenness were also significantly negative for bees. In summary, abundance and the three measures of diversity all varied largely hand‐in‐hand for moths, whereas for bees, abundance and species richness diverged from evenness and the Shannon‐Wiener Index.

**TABLE 4:** Correlations among dependent variables: Abundance, species richness, Shannon‐Wiener Index, and evenness, separately for bees and moths.

	Abundance	Richness	S‐W diversity (*H*)	Evenness
Abundance		+0.90***	+0.91***	0.00
Richness	+0.64***		+0.91***	0.18
S‐W diversity (*H*)	−0.43***	0.25		+0.54***
Evenness	−0.79***	−0.41*	+0.73***	

*Note: R*‐values for bees are shown in orange shading, those for moths in yellow. Positive correlations are shown with ‘+’, negative ones with ‘−’. Significant correlations are bolded, with an indication of statistical significance (*p < 0.05, ***p < 0.001). Degrees of freedom were 28 for bees and 43 for moths.

We calculated correlations using 30 sample sites for bees and all 45 for moths to conform to the “expanded model” and “base model” used for random forest models of bees and moths, respectively. Using all 39 sample sites for bees instead produced slightly different results: no relationship between abundance and *H* and a significantly positive correlation between richness and *H* (results not shown).

#### 3.2.2 Random Forest Models of Relationships of Abundance and Diversity With Environment

**FIGURE 2:**
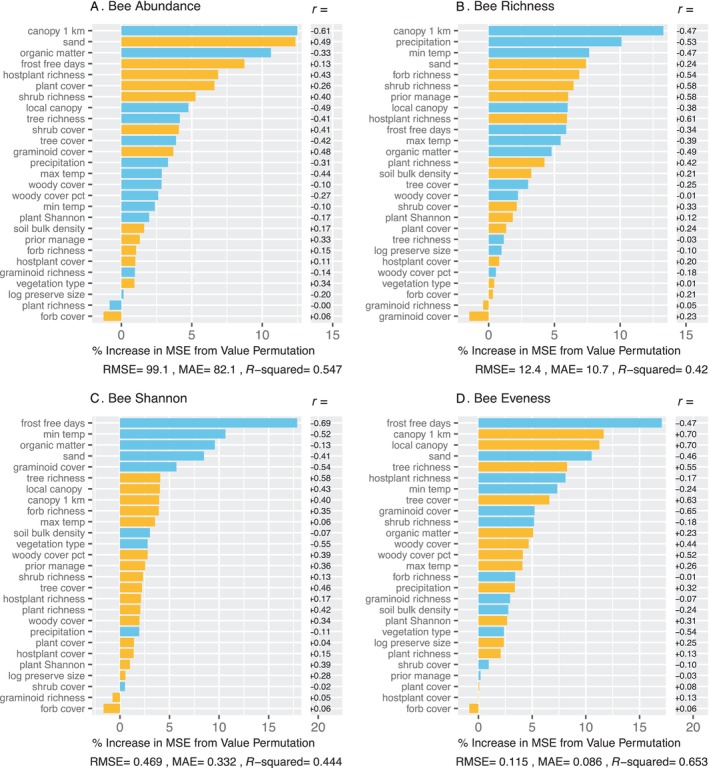
Random forest models for bees, showing importance of predictors of abundance (A), species richness (B), Shannon‐Wiener Index (C), and evenness (D). For each model, root mean square error, mean absolute error, and *R* ^2^ are provided. Predictor importance (percent increase in mean square error from permutation of values) is indicated by the length of the line. Blue lines indicate negative effects; yellow lines indicate positive effects. The effect directions were determined external to the random forest using linear correlations (*r*), which are shown in the column to the right. Climate, canopy, and soil were key predictors for bees, similar to moths (Figure 2), but the directions of the predictor effects and the bivariate plots (Figure 3) reveal strong contrasts with moths. Vegetation, including bee hostplants, was also important to bees. There were large differences in the directions of predictor effects between abundance and species richness versus Shannon‐Wiener Index and evenness.

**FIGURE 3:**
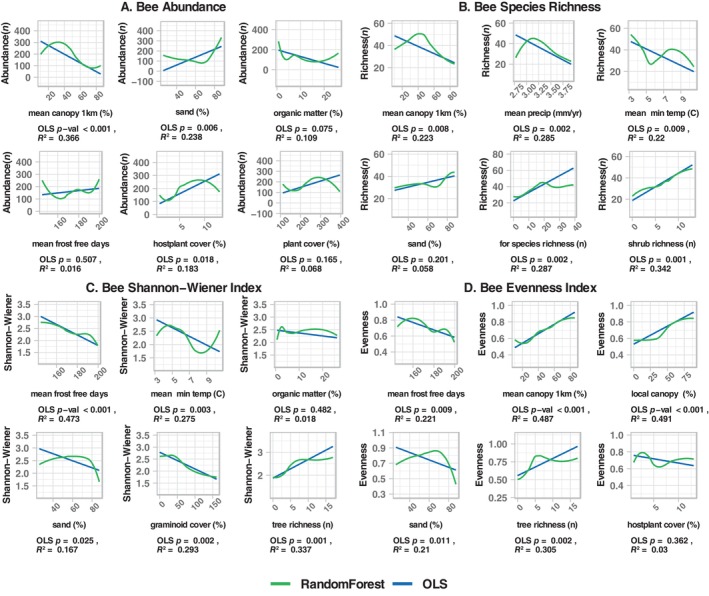
Bivariate plots for the top six predictors from random forest models of bee abundances. Graphs are for relationships between the six top predictors for abundance (A), species richness (B), Shannon‐Wiener Index (C), and evenness (D). For each plot, the random forest fit is shown in green and the ordinary least squares fit in blue. Below each plot, the *p*‐ and *R* ^2^‐values are provided for the linear fit. For bees, abundance and richness were higher in sites with sandy soil, low canopy cover, and some combination of high cover and richness of plant species in lower strata. Shannon‐Wiener Index and evenness largely responded to predictors in an opposite manner.

For bees, the expanded random forest models, which included all predictor variables, performed better than did the base models, despite fewer sample sites (30 vs. 38; Table 5). Across the four dependent variables, aspects of climate, plant cover, and soils were key model predictors (Figure 2). There were differences, however, in the direction of these effects among the response variables. Abundance and species richness were higher on warmer sites, sandy soils with low organic matter, open canopies with a relatively dense low vegetation layer, and high levels of bee hostplant richness (Figures 2A,B and 3A,B). In contrast, bee Shannon‐Wiener Index (*H*) was higher in colder sites with less sand and higher woody canopy cover (Figures 2C,D and 3C,D).

**TABLE 5:** Results of random forest (RF) models for bees and moths, including sample sizes, RSME and *R* ^2^ for two different models.

Predictor set	Year – *N*	RF abundance		RF richness		RF Shannon	
		*R* ^2^	RMSE	*R* ^2^	RMSE	*R* ^2^	RMSE
Bees—Base	2018 – 14 2019 – 21 2020 – 38 2021 – 38	0.68	95.9	0.47	7.7	0.40	0.5
Bees—Expanded	2018 – 06 2019 – 14 2020 – 30 2021 – 30	0.61	107.1	0.51	7.2	0.55	0.5
Moths—Base	2021 – 45 2022 – 45	0.43	354.0	0.63	40.2	0.56	0.4
Moths—Expanded	2021 – 25 2022 – 12	0.13	571.4	0.24	69.2	0.63	0.3

*Note:* The models are the base and expanded RF models. Base = predictor variables excluding field‐collected vegetation variables; expanded = all predictor variables. Separate models were run for each of three different dependent variables: Abundance, species richness, and Shannon‐Wiener Index. See Table 2 for details.

For moths, the base random forest models performed much better overall than did the expanded models (Table 5). This is not surprising given that there were far more sample sites for the base (45) than the expanded models (25) (Tables 2 and 5). Accordingly, for analyses of the relative importance of different predictor variables, we show the results only for the base models for moths.

**FIGURE 4:**
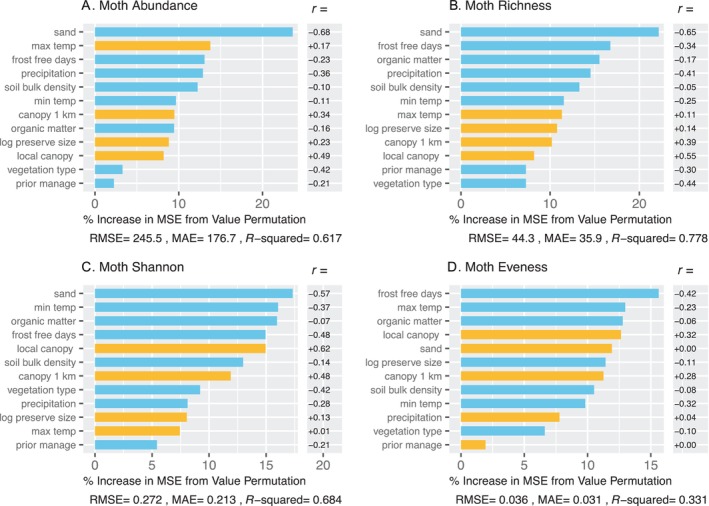
Random forest models for moths, showing importance of predictors for abundance (A), species richness (B), Shannon‐Wiener Index (C), and evenness (D). For each model, root mean square error, mean absolute error, and *R* ^2^ are provided. Predictor importance (percent increase in MSE from permutation of values) is indicated by the length of the line. Blue lines indicate negative effects; yellow lines indicate positive effects. The effect directions were determined external to the random forest using linear correlation (*r*), which are shown in the column to the right. Climate, canopy cover, and soil were the most important predictors for moth abundance and diversity.

**FIGURE 5:**
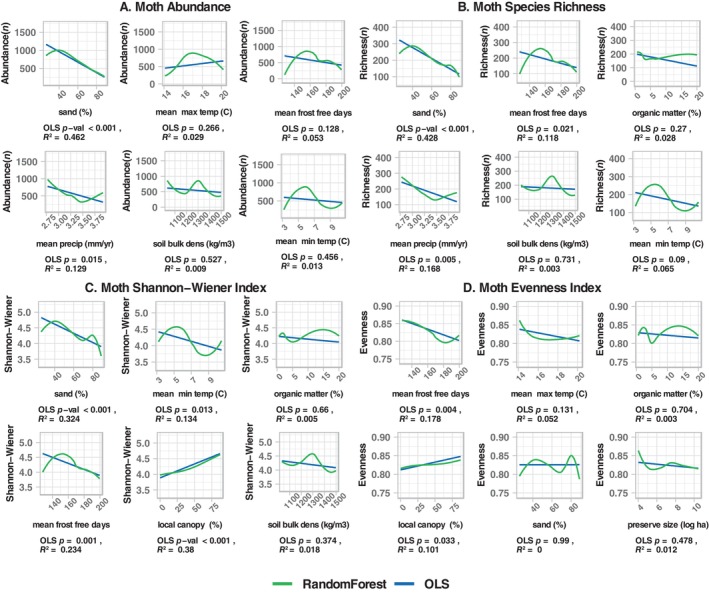
Bivariate plots for the top six predictors from random forest models for moths. Graphs are for relationships between the six top predictors (see Figure 4) for abundance (A), species richness (B), Shannon‐Wiener Index (C), and eveness (D). For each plot, the random forest fit is shown in green and the ordinary least squares fit in blue. Below each plot, the *p*‐ and *R* ^2^‐values are provided for the linear fit. Moth abundance and diversity were generally higher in sites that were relatively cold and had high canopy cover and low percentages of sand.

Variables related to climate, canopy cover, and soils were the most important predictors of moth abundance and diversity (Figure 4). Abundance, species richness, Shannon‐Wiener Index, and evenness were all higher in sites that were colder, experienced a shorter growing season, exhibited denser canopy cover, and were underlain by soils with less sand, higher bulk density, and lower organic matter (Figure 4). These sites are more similar to the mesic landscape of the Northeast USA than are sandier, more open barrens sample sites. Bivariate plots of the six most important predictors for each of the three dependent variables illustrate these patterns in detail (Figure 5). Some of the pollinator‐environment relationships appeared to be largely linear, whereas others exhibited more complex patterns.

**FIGURE 6:**
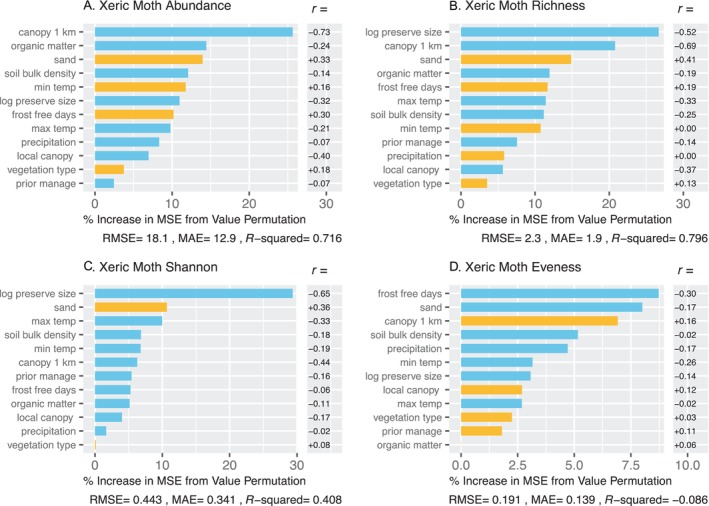
Random forest models for moths associated with xeric habitats for abundance (A), species richness (B), (C) Shannon‐Wiener Index, and eveness (D). For each model, root mean square error, mean absolute error, and *R* ^2^ are provided. For each model, predictor importance is indicated by the length of the line. Blue lines indicate negative effects; yellow lines indicate positive effects. The effect directions were determined external to the random forest using linear correlations (*r*), which are shown in the column to the right.

**FIGURE 7:**
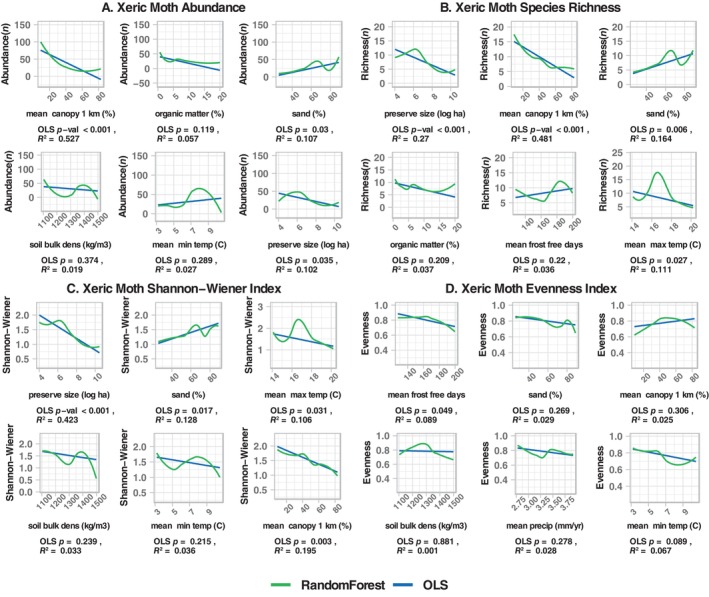
Bivariate plots for the top two predictors from random forest models for presumed xeric‐associated moths for abundance (A), species richness (B), Shannon‐Wiener Index (C), and eveness (D). For each plot, the random forest fit is shown in green and the ordinary least squares fit in blue. Below each plot, the *p*‐ and *R* ^2^‐values are provided for the linear fit. In contrast to the entire set of moths, xeric‐associated moths were more abundant and diverse in sites with less canopy cover and sandier soil, as predicted.

Separate random forest analyses for potential xeric specialist moth species (Table 5) contrasted strongly with the results for all moths. These moths exhibited higher abundance, species richness, and Shannon‐Wiener Index in more open and sandier sites; predictor variables related to openness and percent sand were some of the most important in the random forest results (Figures 6 and 7).

#### 3.2.3 NMDS Analyses of the Relationships of Species Composition With Environment

**FIGURE 8:**
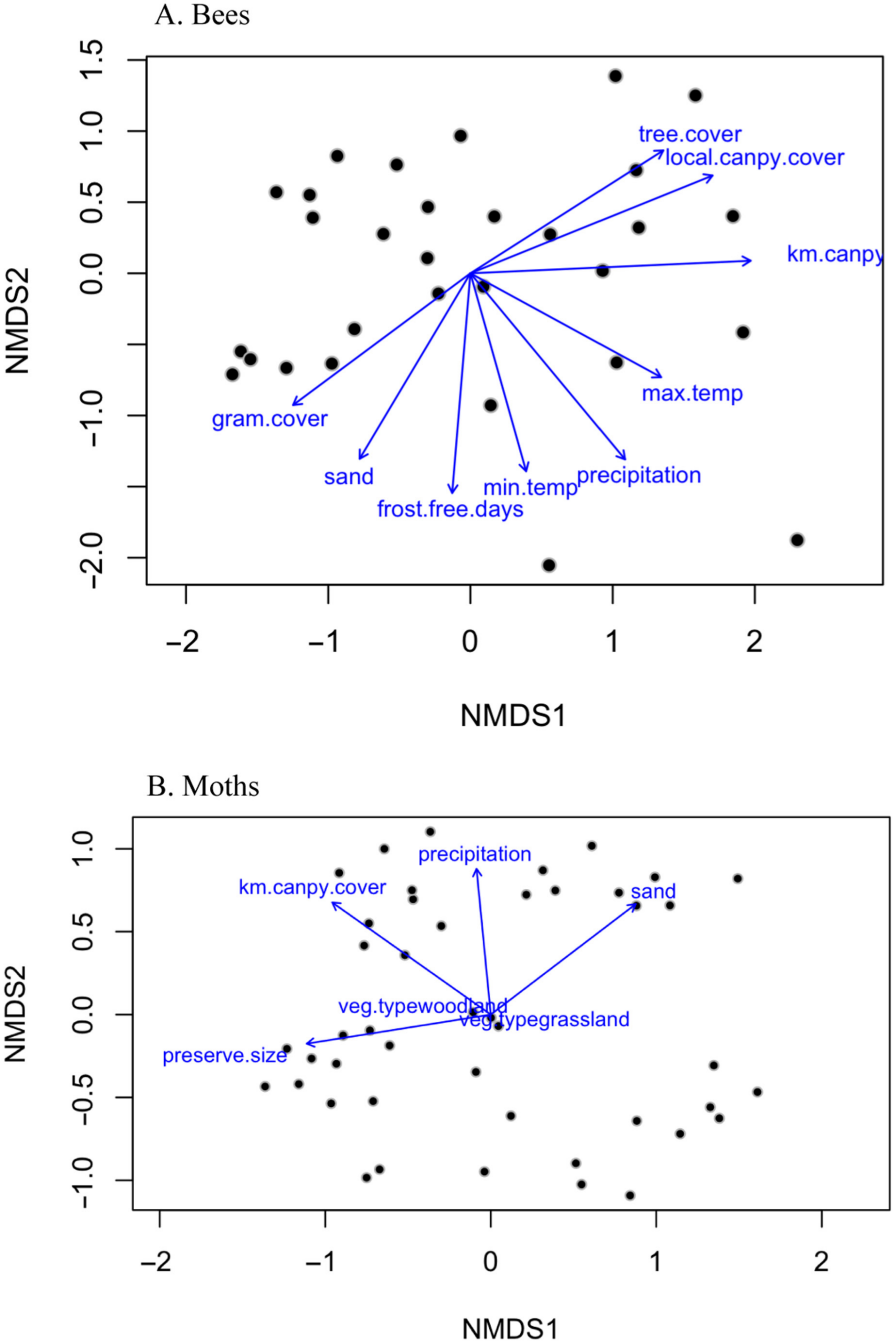
First two axes for non‐metric multidimensional scaling analysis for the species composition of moths (A) and bees (B). Each point is a sample site. Fitted vectors show the direction and strength of the linear correlations (all *p* < 0.05) of the predictor variables with the plot scores. Predictors are described in Table 3. Moth species composition is most strongly correlated with canopy cover, percent sand in the soil, precipitation, and maximum temperature. Key correlates of bee species composition gradients were tree cover, grass cover, percent sand, temperature, and precipitation.

The nMDS solution for bee species produced a stress level of 0.14. The analysis using all predictors (i.e., the expanded model) revealed significant associations of bee species composition with tree and grass cover, climate, and percent sand for the first two axes of the ordination (Figure 8A). These were similar to the most important predictors in the random forest models for bees. The non‐metric multidimensional scaling (nMDS) solution for moth species abundance data produced a stress level of 0.17. The analysis using the predictors for the base model suggested that canopy cover, percent sand, and climate (Figure 8B) were strongly associated with moth species composition. These results are similar to those described previously for the random forest models for moth abundance and diversity.

### 3.3 Joint Analyses of Bees and Moths

**FIGURE 9:**
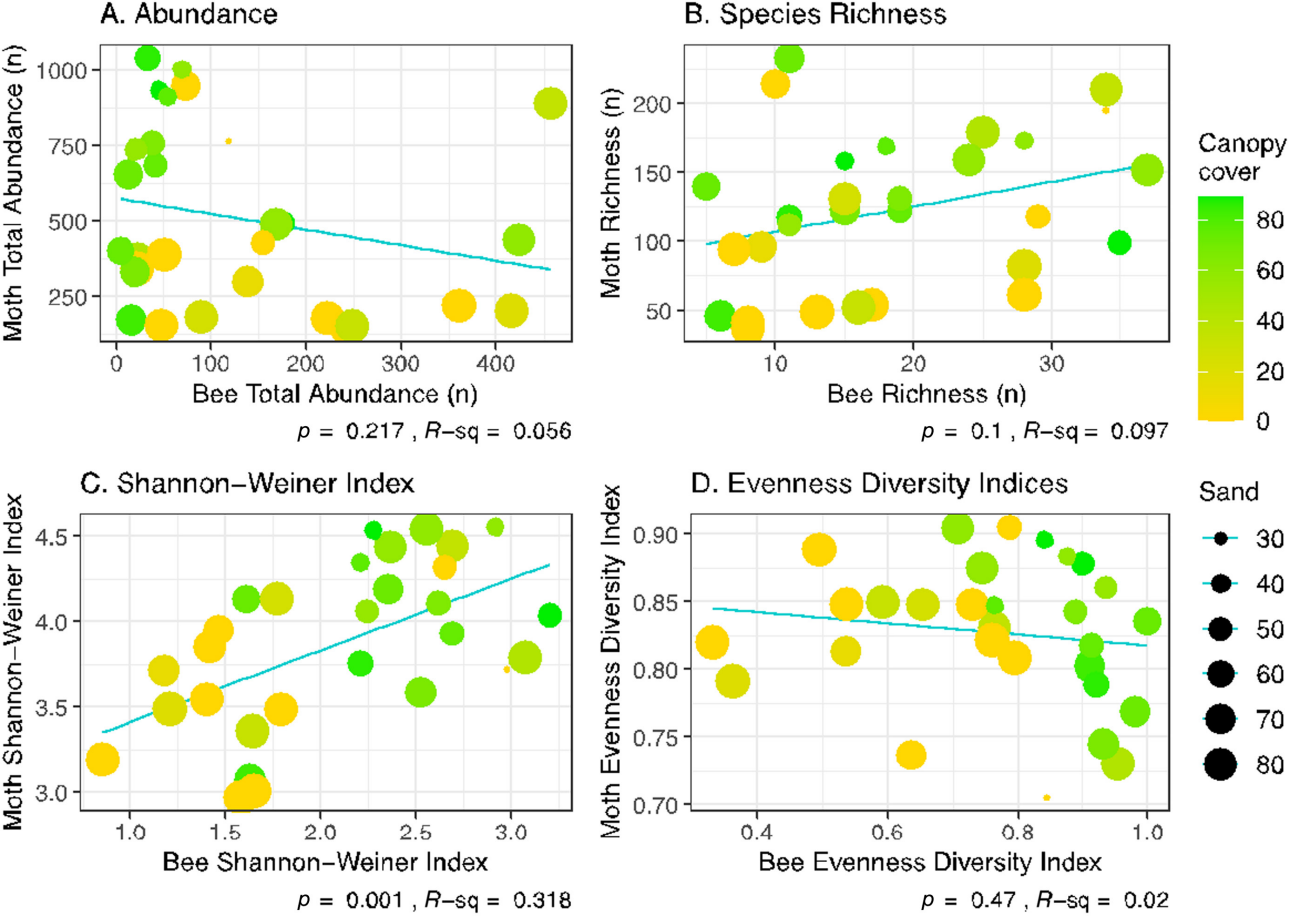
Correlation between bees and moths for abundance (A), species richness (B), Shannon‐Wiener Index (C), and evenness (D). Each point is one sample site; lines, *r*, and *p*‐values are from linear correlations. Relative amount of canopy cover and percent sand are indicated for each sample site. The site used for this analysis represent the subset of all sites in which both bees and moths were sampled.

The relationship between moth and bee abundance was complex and was poorly described by a simple linear correlation analysis, which was not significant (Figure 9A). It's clear that where bees were abundant, moths were not and vice versa, with the exception of the Albany Pine Bush Alley Cat sample site. Where bees were less common, moth abundance varied substantially from very low (148) to very high values (1153). This abundance pattern is illuminated by displaying abundance and diversity with respect to sample site canopy cover and percent sand (Figure 9A). Where moths were abundant, canopy cover was high and percent sand low; where bees were abundant, the opposite conditions prevailed. These results reinforce the random forest models on bee and moth abundance drivers.

Bees and moths exhibited no significant relationship for species richness (Figure 9B), and inclusion of canopy cover and percent sand did not add interpretation to the point scatter. In contrast, bees and moths were positively correlated for the Shannon‐Wiener Index (Figure 9C). Sample sites with high *H* values for both bees and moths clearly exhibited higher canopy cover and lower percent sand compared to those with low values for both. It's notable that in these more forested, less sandy sample sites, bee *H* and evenness were both high and abundance low.

## 4 Discussion

Barrens in the Northeast USA offer unique xeric conditions within a largely mesic, forested landscape. These sites derive a constellation of unusual attributes from an underlying substrate of either sandy soil or exposed rock. The unproductive soils, coupled with natural or anthropogenic disturbance, constrain vegetative cover, promoting open canopies (Bois et al. 2023; Bried, Patterson III, and Gifford 2014; Dunwiddie 1998; Foster and Motzkin 2003; Motzkin and Foster 2002). Our results corroborate prior studies by demonstrating the importance of these unique conditions for supporting a diverse assemblage of bees and moths, including regionally and globally rare species (Bois et al. 2023; Bried, Patterson III, and Gifford 2014; Bried and Gifford 2010; Drummond et al. 2017; Raleigh, Capece, and Berry 2003; Selfridge et al. 2017; Tucker and Rehan 2019; Wagner, Nelson, and Schweitzer 2003).

A major result of our study is that communities of bees and moths contrast in their response to two key environmental drivers: canopy openness and percent sand. Bee abundance and species richness were highest in more open sites with sandier soil, whereas moths exhibited the opposite pattern. Other studies have also found that bee abundance and diversity are higher in more open barren vegetation in the Northeast (Bried and Gifford 2010; Tucker and Rehan 2019) and elsewhere (Campbell, Hanula, and Waldrop 2007; Campbell et al. 2018; Gelles et al. 2021; Grundel et al. 2010; Kalhorn, Barrows, and LaBerge 2003; Lettow et al. 2018; Odanaka et al. 2020; Ulyshen et al. 2022). Across the globe, in fact, the abundance and species richness of bees are generally higher in more open sites, which usually support a relatively high density of flowers (Antoine and Forrest 2021; Hanula, Ulyshen, and Horn 2016; Michener 2000). Relationships of moth communities to vegetation conditions are more complicated and less well‐known than for bees (Anderson, Rotheray, and Mathews 2023; Bried and Gifford 2010; Hahn and Brühl 2016; Schoppmann 2016).

The contrast in the responses of bees and moths to canopy openness and percent sand is consistent with differences in life history between the two groups. Most bee species nest in the ground, where juveniles develop to adulthood (Antoine and Forrest 2021; Harmon‐Threatt 2020). More porous soils, such as sand, provide ideal nesting conditions for these ground nesters. Most adult bees feed themselves and their brood with nectar or pollen collected from flowers, primarily of forbs and shrubs (Antoine and Forrest 2021; Fowler 2016; Hanula, Ulyshen, and Horn 2016; Michener 2000). The abundance of these food resources tends to be higher in places with relatively low tree cover. It is not surprising, therefore, that barrens sites with sandier soil and more open conditions support higher levels of bee abundance and species richness. Like for bees, adult moths readily forage on flowers (supplying nectar), but many moth species lay their eggs on woody plants, including those creating canopy cover in our study areas, where their larvae develop into pupae and adults (Grand and Mello 2004; Lintott et al. 2014; Maier 2004; Schoppmann 2016; Wagner, Nelson, and Schweitzer 2003). Thus, although more open conditions can potentially supply moths with high levels of adult resources, this might be inversely related to juvenile food supply for some moth species. This complex life history, therefore, may account for lower levels of overall moth abundance and species richness in open, sandy sites. In northeastern barrens, in particular, some moth species use scrub oak (*Quercus ilicifolia*) as a larval host, and their abundance and richness are, therefore, likely tied to the density of this oak species host (Bried and Gifford 2010; Grand and Mello 2004; Schoppmann 2016; Wagner, Nelson, and Schweitzer 2003). Oaks constituted a mean of 19.3% (range = 0–71) of total plant cover, and oak and total cover were positively correlated (*t* = 2.9, *p* < 0.01, *r* = 0.13) in our samples. Therefore, low levels of moth abundance and richness under low canopy cover might have been in part a result of lower availability of oak host plants.

The results also reveal, however, that moths captured in our barren sites were not homogeneous in their response to canopy openness and percent sand. The subset (69 out of 921) of potentially xeric‐specialist moth species responded similarly to bees rather than to the other moths. It is highly likely that both bees and moths using northeastern barrens vary among species in their habitat preferences regarding soils and canopy cover. In sites other than these barrens, in fact, dense forest cover and different forest management practices support different functional groups of bees (Chase et al. 2023; Smith et al. 2021). The stark contrast between bees and moths revealed in our results likely stakes out two poles of a spectrum of responses to these habitat conditions, with more bee species selecting more open and sandy conditions and more of the detected moth species preferring the opposite habitat conditions. These and other patterns described here are, of course, subject to the caveat that we can only report results for those bee and moth species that were captured during the study.

In sharp contrast to the differing trends between bees and moths for abundance and species richness, these two groups exhibited similar responses for the Shannon‐Wiener Index (*H*). For both, *H* increased from less sandy, more open sites to more closed, less sandy sites. For moths, both richness and evenness increased across this gradient, jointly leading to higher *H*. The pattern for bees is more complex. Bee species richness and abundance declined across the gradient, and increasing evenness was the sole driver of the rise in *H*. An examination of the responses of individual species abundance illuminates these patterns of richness, evenness, and *H* for bees. Higher bee abundance in open, sandy sites, which led to higher richness, was largely the result of a disproportionate numerical increase in a very small subset of species. This pattern substantially decreased evenness in those sites—and as a consequence also reduced the Shannon‐Wiener index. In fact, the percentage of total bee abundance accounted for by the most common bee, *Augochlorella aurata* (Smith, 1853; Halictidae), a eusocial ground‐nesting sweat bee (Ordway 1964), increased significantly with increased total bee abundance across sites (Spearman Rank Test: *r*_s_ = 0.65, *t* = 1.9, *p* < 0.0001). Notably, this species reached > 75% dominance in sites with the highest overall bee abundance. In other words, open, sandy sites supported high numbers of bees, but most were of one or few species—very low evenness. Similar disproportionate abundances across sites were not found for moths. This divergence for bees in abundance and species richness vs. evenness and Shannon‐Wiener Index resulted, therefore, in a positive correlation in Shannon‐Wiener Index between bees and moths.

The contrast between bees and moths in the relationship of species richness and Shannon‐Wiener Index raises an important question: how do these two metrics differ in their relevance to pollinator conservation? Species richness is a simple count that gives equal weight to rare and abundant species. From the perspective of species viability, weighing species equally is critical, for even small populations contribute to population redundancy within a species, reducing the probability of extinction from disturbances and demographic stochasticity (Shaffer and Stein 2000). With its inclusion of evenness, the Shannon‐Wiener Index downweights rare species but is a better measure than richness of the actual species variation in pollinator visitation experienced by plant species. In this sense, the Shannon‐Wiener Index provides a more relevant functional estimate of community‐level plant‐pollinator species interactions. Our results suggest that more bee species are supported at the more open, sandier end of a barren gradient, whereas moths exhibit the opposite pattern. In contrast, bees and moths both provide more variety in pollination services in less open and less sandy barren sites. At least considering only bees and moths, it is possible, therefore, that a broader variety of plant species are provided with adequate pollination services in these less extreme barren sites with more canopy cover and less sandy soils—places that combine the attributes of unique barren environments with more mesic, forested sites. These patterns also emphasize the importance of examining both species richness and diversity indices, such as the Shannon‐Wiener Index, that include evenness when evaluating community‐level ecological patterns and the impacts of management actions.

Our results have important implications for the management of pollinators in barren sites. Some of the key predictors in these analyses, such as climate, are not under local management control, whereas others, such as vegetation structure and plant composition, both of which matter to these pollinators, can be altered by habitat manipulations. The contrasting habitat preferences of bees and moths suggest that managing for spatial heterogeneity is likely to promote the highest abundance and species diversity of both bees and moths. Barrens already contribute heterogeneity to the largely mesic landscape of the Northeast USA and, in so doing, support higher diversity of these taxa, especially for rare bee and moth species. Our results suggest that the presence of both closed and open canopies at the finer‐grained scale of barrens themselves will have a similar positive impact on biodiversity. Several studies have provided evidence favoring a patch mosaic approach to habitat management in northeastern barrens for vegetation (Jamison, D'Amato, and Dodds 2023), bees and moths (Bried and Dillon 2012; Grand and Mello 2004; Schoppmann 2016; Wagner, Nelson, and Schweitzer 2003), and ants (Bradford 2023). Similar support for a managing habitat heterogeneity has been found for insects elsewhere (Engle et al. 2008; Gelles et al. 2021; Grundel et al. 2010; Smith et al. 2021; Ulyshen et al. 2022), although the patch dynamics approach has been criticized for its overly broad application to insects and vertebrates (Parr and Andersen 2006). Therefore, although managing for a variety of conditions might not promote abundance and diversity of all target groups of organisms in all locations, our results strongly suggest that it does for bees and moths, although this recommendation comes with the caveat that not all xeric bee and moth life histories are well understood. Given that these results may emerge from fundamental differences in life histories of bees and moths, these management implications are likely to apply to barren habitats in other parts of the world.

Over the last two decades, a groundswell of support has emerged for active management of barren sites in the Northeast USA and elsewhere because they support a diversity of species, including rare and endemic taxa, less represented in the more mesic surrounding landscape (Bois et al. 2023; Bried, Patterson III, and Gifford 2014; Jamison, D'Amato, and Dodds 2023; Littlefield and D'Amato 2022; Wagner, Nelson, and Schweitzer 2003). Since the 1980s, conservation has embraced a coarse‐filter/fine‐filter approach to sustaining biodiversity (Noss 1987). Coarse filters focus on elements of the environment that, if maintained, will sustain a diversity of species, communities, and ecosystem processes. Fine filters attend to species for which coarse filters are insufficient to meet requirements for their viability. Initially, coarse filters targeted communities, although it was acknowledged that communities are impermanent and, thus, merely a means to an end. Hunter Jr, Jacobson Jr, and Webb III (1988) (see also Anderson, Clark, and Sheldon 2014) advocated a more direct coarse filter approach based on physical locations that serve as current and likely future hotspots for biodiversity. As rare substrates that support xeric habitats in an otherwise mesic landscape, barrens are a clear example of such physical arenas that sustain ecological richness and habitat diversity. As environments change with continued anthropogenic pressures, barrens are likely to continue to protect an important range of species not accommodated elsewhere, even if the actual communities inhabiting these sites change over time. This suggests that active management of such rare sites, directed at maintaining their unique conditions, should play an important role in conservation.

## Author Contributions

**Andrew M. Barton:** conceptualization (lead), data curation (lead), formal analysis (lead), investigation (lead), methodology (equal), validation (equal), visualization (equal), writing – original draft (lead), writing – review and editing (lead). **Helen M. Poulos:** conceptualization (lead), data curation (lead), formal analysis (equal), investigation (equal), methodology (equal), validation (equal), writing – original draft (equal), writing – review and editing (equal). **Elizabeth Crisfield:** conceptualization (lead), data curation (lead), funding acquisition (lead), investigation (supporting), methodology (supporting), project administration (lead), supervision (lead), writing – review and editing (equal). **Amanda Dillon:** conceptualization (equal), data curation (equal), investigation (lead), methodology (lead), project administration (supporting), resources (supporting), supervision (equal), validation (equal), writing – review and editing (supporting). **Mark Mello:** conceptualization (equal), data curation (equal), investigation (lead), methodology (lead), project administration (supporting), supervision (supporting), validation (equal), writing – review and editing (supporting). **Jennifer Selfridge:** conceptualization (equal), data curation (equal), investigation (lead), methodology (lead), project administration (supporting), resources (supporting), supervision (equal), validation (equal), writing – review and editing (supporting). **Rick Van de Poll:** conceptualization (supporting), data curation (supporting), investigation (lead), methodology (lead), project administration (supporting), supervision (supporting), writing – review and editing (supporting). **Sarah Hardy:** data curation (equal), formal analysis (equal), investigation (supporting), visualization (equal), writing – review and editing (supporting).

## Ethics Statement

The authors have nothing to report.

## Consent

Consent by all authors is given for publication.

## Conflicts of Interest

The authors declare no conflicts of interest.

## Supporting information


Table S1.

## Data Availability

Reviewers can view the data at https://datadryad.org/stash/share/sIZ7ySkoRQkcesFTRpF3VjYFCUnYLky3DlQMSz4_‐Og. After publication, the data will be publicly available at https://doi.org/10.5061/dryad.n5tb2rc3z.
