# Supplementary material for: Moths versus Bees: Contrasts in Habitat Preferences Across Barrens of the Northeastern USA
Source: Ecol Evol. 2024 Nov 13;14(11):e70533. doi: 10.1002/ece3.70533 (PMC11560292; doi:10.1002/ece3.70533)
Supplement: Supplementary file 1 — Table S1. [file ECE3-14-e70533-s001.docx]

**SUPPLEMENTS**

Table S1. Xeric specialist moth species.

| *Abagrotis benjamini* | *Dasylophia anguina* | *Properigea costa* |
| --- | --- | --- |
| *Abagrotis brunneipennis* | *Datana contracta/integerrima* | *Psectraglaea carnosa* |
| *Acronicta tritona* | *Drasteria graphica* | *Schinia septentrionalis* |
| *Agrotis cf. buchholzi* | *Drasteria occulta* | *Sideridis maryx* |
| *Amolita roseola* | *Eacles imperialis* | *Sideridis rosea* |
| *Apamea burgessi* | *Erastria coloraria* | *Spilosoma dubia* |
| *Apamea inordinata* | *Euchlaena madusaria* | *Stenaspilatodes antidiscaria* |
| *Apantesis anna* | *Eucoptocnemis fimbriaris* | *Sympistis dentata* |
| *Apodrepanulatrix liberaria* | *Eueretagrotis attentus* | *Sympistis riparia* |
| *Catocala badia* | *Eumacaria madopata* | *Syngrapha epigaea* |
| *Cerma cora* | *Euxoa perpolita* | *Xestia elimata* |
| *Chaetaglaea cerata* | *Euxoa violaris* | *Xylena thoracica* |
| *Chaetaglaea rhonda* | *Feltia manifesta* | *Xylotype capax* |
| *Chaetaglaea tremula* | *Gabara subnivosella* | *Zale buchholzi* |
| *Chytonix sensilis* | *Heterocampa varia* | *Zale curema* |
| *Cicinnus melsheimeri* | *Hyparpax aurora* | *Zale lunifera* |
| *Cingilia catenaria* | *Hyperstrotia flaviguttata* | *Zale squamularis* |
| *Cisthene packardii* | *Lapara coniferarum* | *Zale submediana* |
| *Cleora projecta* | *Macaria exonerata* | *Zanclognatha martha* |
| *Coelodasys apicalis* | *Meropleon ambifusca* |  |
| *Crambidia xanthocorpa* | *Metarranthis pilosaria* |  |
| *Cucullia speyeri* | *Morrisonia mucens* |  |
| *Cyclophora culicaria* | *Nepytia pellucidaria* |  |
| *Cycnia collaris* | *Pelochrista adamantana* |  |
| *Dasychira pinicola* | *Phoberia ingenua* |  |
